# The optimal calibration hypothesis: how life history modulates the brain's social pain network

**DOI:** 10.3389/fnevo.2012.00010

**Published:** 2012-07-05

**Authors:** David S. Chester, Richard S. Pond, Stephanie B. Richman, C. Nathan DeWall

**Affiliations:** Department of Psychology, University of Kentucky, LexingtonKY, USA

**Keywords:** social pain, social rejection, life history, attachment style, anterior cingulate cortex, anterior insula

## Abstract

A growing body of work demonstrates that the brain responds similarly to physical and social injury. Both experiences are associated with activity in the dorsal anterior cingulate cortex (dACC) and anterior insula. This dual functionality of the dACC and anterior insula underscores the evolutionary importance of maintaining interpersonal bonds. Despite the weight that evolution has placed on social injury, the pain response to social rejection varies substantially across individuals. For example, work from our lab demonstrated that the brain's social pain response is moderated by attachment style: anxious-attachment was associated with greater intensity and avoidant-attachment was associated with less intensity in dACC and insula activation. In an attempt to explain these divergent responses in the social pain network, we propose the *optimal calibration hypothesis*, which posits variation in social rejection in early life history stages shifts the threshold of an individual's social pain network such that the resulting pain sensitivity will be increased by *volatile* social rejection and reduced by *chronic* social rejection. Furthermore, the social pain response may be exacerbated when individuals are rejected by others of particular importance to a given life history stage (e.g., potential mates during young adulthood, parents during infancy and childhood).

*Pain is as diverse as man. One suffers as one can*.

— Victor Hugo

Social rejection hurts. Indeed, instances of rejection activate the same brain regions as physical pain (Eisenberger et al., 2003). This phenomenon is not mysterious, given the pivotal role of group membership in human survival and reproductive outcomes. However, if an individual faced rejection on a daily basis for an extended period of time, would such chronic pain still be useful? Would someone who experienced rejection in an unpredictable, chaotic manner profit from a pain system that was sensitive to such variability in social bonds? In the present article, we argue that in either case, the individual would stand to lose more than they gained from an immutable, static social pain system. To effectively respond to the natural variation in human social ecology, an individual would require a dynamic, flexible social pain system. Drawing from the vast literature on attachment styles, we posit that the sensitivity of the social pain system is flexible in infancy and childhood, but stabilizes in adolescence and adulthood. As such, we put forward the *optimal calibration hypothesis*, which posits that the frequency and intensity of social rejection in early life history stages (i.e., infancy, early childhood) influence how much the brain's social pain network responds to social rejection in later life history stages (e.g., adulthood). The resulting changes in pain sensitivity in adulthood will represent that individual's social ecology in infancy and childhood. More specifically, we hypothesize that *chronic* social rejection during early life history stages will predict a less sensitive adult social pain network while *volatile* social rejection at this same time will result in a more sensitive adult social pain network. The present article does not serve to empirically test either of these hypotheses, instead laying bare the theoretical rationale behind their formation and potential implications for extant theory.

In the present article, we begin by reviewing the literature on social pain and its neural correlates. Second, we couch our theoretical model in the literature on attachment style, arguing that early-life experiences of rejection are the principal causes of calibration in social pain. Third, we review fitness benefits derived from calibrating the social pain network. Finally, we extend the optimal calibration hypothesis to all life history stages, putting forth testable predictions that the social pain network is sensitive to the source of rejection, responding preferentially to rejecters of utmost importance to a given life history stage.

## Social pain and the need to belong

People are driven to seek out and maintain positive relationships with others through a fundamental need to belong, which is pervasive across time and cultures (Baumeister and Leary, 1995). This fundamental motivation toward belongingness is deep-seated in human evolutionary history. Early hunter-gatherers were not suited for solitary life and, thus, formed supportive, communal bands with others to fulfill many basic needs of survival (Buss, 2008). Like most eusocial species, early humans depended on reciprocal altruism in the form of group efforts toward obtaining food stores, providing shelter, and defending against bodily harm (Trivers, 1971). Moreover, compared to other mammals, human beings are constrained by an extended infancy in which critical brain development occurs outside of the womb. By forming parental bonds and sharing childcare responsibilities, early hunter-gatherers were able to ameliorate the survival burdens placed on mothers and their infants during this prolonged period of vulnerability (Eastwick, 2009).

Within the context of early human history, enduring a socially painful event (e.g., being shunned or ostracized) could be as detrimental to survival as physical injury. Thus, those early humans who had a greater capacity for maintaining group membership were better equipped for surviving and passing on their genes to subsequent generations compared to their less sociable counterparts. Because social threats, such as rejection, rivalries, and any other loss of social status or group membership were costly in terms of survival and reproduction, psychological mechanisms protecting against social threats evolved (Leary and Downs, 1995; Kurzban and Leary, 2001; Leary, 2001). Critical among these psychological mechanisms was co-opting the physical pain system for signaling social threats (Panksepp, 1998; Eisenberger and Lieberman, 2004; MacDonald and Leary, 2005; Eisenberger, 2012).

## Overlap of physical and social pain

A substantial body of literature has proposed that evolutionary forces resulted in the co-option of the body's existing physical pain system for responding to socially painful events (e.g., Herman and Panksepp, 1978; Panksepp et al., 1978a,b; Panksepp, 1998). Because social threats posed serious risks to one's survival and reproductive fitness, it was important to monitor social dangers efficiently. Given that the evolution of overlapping neural substrates for physical and social pain would be predicated on the existence of social threats, it is expected that social threats preceded the aforementioned neural overlap in time. Pain is an effective alarm that communicates the presence of danger to an organism (Price, 1988). Thus, a social-attachment system that co-opted the use of neural-cognitive mechanisms already in place for monitoring physical pain would be more efficient and economical than two systems that regulated both physical and social pain separately. One shared pain system should, therefore, reflect similarities in the ways that physical and social pain are encoded and perceived.

Several lines of research support the theoretical model that physical pain and social pain each are encoded and perceived through similar neural structures that signal injury (see MacDonald and Leary, 2005 for a review). First, there is a linguistic similarity in the terms people use to describe physically and socially painful events. For instance, when vividly recalling a past episode of social rejection, one might say that he or she felt “hurt” or “crushed” (Leary and Springer, 2001). Moreover, the linguistic similarity between physical and social pain is not a product of Western culture, because people associate social pain with physical pain in many languages across the globe, including German, Hebrew, Mandarin, and Inuktitut, as well as at least 10 others (MacDonald and Leary, 2005).

Second, DeWall and Baumeister (2006) tested whether social rejection affected physical pain sensitivity in humans. They found that when people believed that they would end up alone later in life, their pain threshold and tolerance significantly increased compared to non-rejected people. These findings have since been replicated (Borsook and MacDonald, 2010; Bernstein and Claypool, 2012). This suggests that social pain can cause people to become numb to physical pain, which is likely due to their shared neural substrates.

Third, at both the cognitive and behavioral levels, responses to social pain tend to mirror responses to physical pain. For example, experiencing social rejection increases aggressive behavior (e.g., Leary et al., 2003; Twenge and Campbell, 2003; Buckley et al., 2004; DeWall et al., 2009; see Leary et al., 2006; for a review). In the same manner, a large body of evidence has shown that physical pain stimuli increase aggressive responding in humans (e.g., Berkowitz et al., 1981; Berkowitz and Thome, 1987; Giancola and Zeichner, 1997; Giancola, 2003). Social rejection causes people to shift their attention to stimuli that will reduce the pain of rejection, such as signs of social acceptance (e.g., Gardner et al., 2000; Pickett et al., 2004; Maner et al., 2007; DeWall et al., 2009; DeWall, 2010). Similarly, physical pain causes people to fixate their attention on stimuli that are linked to safety and security (Aldrich et al., 2000). The similarity between physical and social pain runs deeper than verbal descriptors or behavioral responses. The next section reviews evidence regarding a neurbiological overlap between social and physical pain.

## The social pain network

The social pain network is comprised of several subcortical regions conserved across mammalian evolution (also referred to as the PANIC/GRIEF system; see Panksepp, 2011): the pariaqueductal gray (PAG), dorsomedial thalamus (DMT), stria terminalis, septal and preoptic areas; as well as two neocortical regions: the dorsal anterior cingulate cortex (dACC) and the anterior portion of the insula. The neuroimaging literature indicates that two regions form the central hub of the social pain network: the dACC and the anterior insula.

### Anterior cingulate cortex

The anterior cingulate cortex (ACC) is located in the midline of the frontal lobe, bordered inferiorly by the corpus callosum and superiorly by the medial prefrontal cortex. The ACC is functionally and anatomically divided into dorsal and ventral regions (Allman et al., 2001). A common approach to these divergent portions of the ACC is that the ventral region is associated with emotional processing and the dorsal region (dACC) is associated with cognitive processing. However, research implicating the dACC as the central hub of the social pain network adds nuance to this conceptualization, as social pain includes a strong affective component (see Eisenberger and Lieberman, 2004).

The dACC is a key structure associated with the affective component of pain (see Apkarian et al., 2005 for a review) and functions as a “neural alarm system” for physical threats (Nelson and Panksepp, 1988; Bush et al., 2000; Eisenberger and Lieberman, 2004). However, recent functional magnetic resonance imaging (fMRI) research has indicated that the dACC plays a role in detecting social threats as well. In their seminal study, Eisenberger and colleagues (2003) assessed neural activation while participants played a virtual ball-tossing game with two fictitious partners who were, ostensibly, also in nearby MRI scanners (Cyberball: Williams et al., 2000). In reality, the “partners” were pre-programmed computers. During the first round of the game, participants were accepted (i.e., received a ball toss from one of the virtual players 33% of the time). However, during the second round, the virtual players stopped throwing the participant the ball after seven throws. Participants were rejected for the remainder of the game and watched as the two virtual players continued throwing the ball back-and-forth. As predicted, the dACC was more active during times of rejection than acceptance. The dACC was also the only brain region whose activation in response to rejection corresponded to greater levels of self-reported distress due to the rejection, implicating this neocortical region as the central hub of the social pain network.

### Anterior insula

With strong functional connectivity to the ACC, the insula is a region of cortex located underneath the opercula along the Sylvian fissure that divides the frontal and temporal lobes and contains bidirectional projections to most other regions of the cortex (Reynolds and Zahm, 2005). Functionally, the insula has been characterized as an integrative center for visceral, bodily sensations (e.g., warmth, hunger) which are then given an affective valence (e.g., positive, negative; Craig, 2002). This associative and evaluative role of the insula lead many scholars to posit that it is involved in the formation of human consciousness (Craig, 2011). Eisenberger and colleagues (2003) found that the anterior insula was activated during instances of rejection, which was predicted due to this region's previous association with negative affect (e.g., Lane et al., 1997) and physical pain (e.g., Aziz et al., 2000).

Another functional domain of the insula, which is inherently related to its association with negative affect and visceral sensation, is that of risk, reward and the perception thereof. Preuschoff et al. (2008) reported that bilateral activation in the insula tracked the perceived riskiness of pursuing a potential reward. In a complimentary line of investigation, other scholars have reported that insula activation correlates with the uncertainty of a given reward (Elliott et al., 2000). This nuanced view of the insula implicates it as a brain region inherently involved in learning from environmental cues. As such, the insula appears as a prime candidate through which early social experiences can lead to the calibration of pain responses through changes in perceived risks and rewards.

## Social pain and attachment

Attachment theory is grounded in the idea that people have a fundamental desire to avoid social pain. Adult attachment researchers typically define attachment styles based on two underlying dimensions: anxiety and avoidance (Griffin and Bartholomew, 1994; Brennan et al., 1998; Fraley and Waller, 1998). Anxious attachment refers to the degree to which people worry about being rejected or abandoned by close others. Avoidant attachment refers to the degree to which people limit intimacy with and avoid getting close to others. Secure individuals are low in both anxiety and avoidance; they feel valued by others and are comfortable developing close relationships.

Anxiously-attached individuals appear to have a “hyperactivated” social pain system. They display intensified social emotions which originate from their desire to capture a caregiver's attention, a strong indicator of investment (Cassidy, 1994). As a child, this hyperactivation would lead to adaptive outcomes as it should result in increased attention from the caregiver and thus greater likelihood of survival (Belsky et al., 1991; Belsky, 1997). Even into adulthood, anxious attachment is associated with more intensely negative responses to actual and imagined rejection (Campbell et al., 2005; Besser and Priel, 2009). Conversely, avoidantly-attached individuals appear to display a “deactivated” social pain system, emotionally and even physically distancing themselves from attachment figures (Mikulincer and Shaver, 2003).

These differences in avoidant and anxious attachment styles result from early social ecologies characterized by inadequate care-giving (Mikulincer and Shaver, 2007). Avoidant individuals have often experienced chronic social rejection, stemming from cold caregivers who rejected their needs for comfort and acceptance. Anxiously-attached individuals often have early social ecologies characterized by volatile social rejection, with caregivers showering affection upon them one minute and ignoring them the next. Because such early life experiences are often indicators of what the conditions of adulthood will be like, the calibration of the social pain network during early childhood is durable across time to account for this likelihood that challenges faced early on will be faced again.

If anxious individuals have a truly hyperactivated system of detecting and responding to social rejection, and avoidant individuals have a truly deactivated system, we should be able to see differential activation in regions of the brain associated with social pain.

## Social pain is moderated by attachment style

Corroborating decades of work on attachment styles, recent work from our laboratory has demonstrated that the social pain system is activated differently between anxiously- and avoidantly-attached individuals (DeWall et al., 2012). In this study, participants completed a measure of attachment style and then played Cyberball in an MRI scanner (Williams et al., 2000). As described earlier, Cyberball is a computerized ball-tossing game in which participants are initially included and then excluded from tossing the ball by their fictitious partners. Results showed that during rejection, people higher in anxious attachment had heightened activity in both the dACC and the anterior insula, whereas people higher in avoidant attachment had dampened activity in these areas (DeWall et al., 2012). People higher in anxious attachment appear to have a highly sensitive social pain network, responding strongly to rejection. Conversely, people higher in avoidant attachment appear to have a desensitized social pain network. This finding for avoidantly-attached individuals fits with previous research demonstrating their neural disengagement from social signals of acceptance and rejection. As a prime example, avoidantly-attached individuals have been shown to display a reduced response in the striatum, the reward center of the brain, in response to positive social feedback (Vrtièka et al., 2008).

Attempting to explain the divergence in recruitment of the social pain network between anxiously- and avoidantly-attached individuals was the impetus behind the formation of the optimal calibration hypothesis. We posit that this difference in social pain activation is representative of the calibration the social pain network undergoes in early life. But how did these differences arise in the brain? In the next section, we propose that the ability of the social pain network to be calibrated by the social ecology of early life history has produced a substantial array of benefits that include the recruitment of parental investment, health benefits, and adaptive mating strategies.

## Evolved function of social pain calibration

As we have titled our theoretical model the optimal calibration hypothesis, we assert that there are substantial benefits behind modulating the sensitivity of the social pain network in early life history stages. Pain serves as an informative signal to warn us of threats and injury in our environment. However, both physical and social forms of pain are no longer useful when they are chronic because the signal becomes no longer informative. Avoidant individuals may avoid this issue by down-regulating their social pain sensitivity so that the chronic rejection they experience does not leave them in constant pain. As evidence, avoidant attachment is associated with worse memory of attachment-related events (e.g., intimacy, separation, and loss), a strategy that would protect people higher in avoidant attachment from the pain of rejection (Fraley et al., 2000; Fraley and Brumbaugh, 2007). Meanwhile, anxiously-attached individuals may heighten the sensitivity of their social pain network to increase the detection of the unpredictable rejection that characterized their development.

### Recruitment of parental investment

Aside from the proximal benefits of effectively coping with and detecting instances of rejection, calibration of the social pain network may function to elicit greater investment from caregivers. Both avoidant and anxious attachment styles derive from an environment in which such care-giving resources are scarce, but children have strategies to obtain investment that are tailored to their given social ecology. People high in avoidant attachment often have mothers who both physically and emotionally rejected them as infants (Ainsworth, 1982). When these parents are unwilling to invest in their children, the avoidant strategy serves as a self-protective mechanism from being abandoned or abused (Chisholm, 1996). These defenses block emotional reactions to the unavailability of attachment figures and down-regulate threat-related emotions, which may serve to engender care-giving responses (Mikulincer and Shaver, 2007). As a child, this deactivation of the social pain network would lead to adaptive outcomes as it protects the child from being abandoned by an aloof caregiver (Chisholm, 1996).

When parents are willing but unable to invest in their children, the anxious strategy maximizes available investment by displaying increased signs of need. In this case, the biological payoff is clear for the offspring. An anxiously-attached child excessively seeks proximity to and reassurance from his or her caregiver, a strategy that should result in increased attention from the caregiver and thus greater likelihood of survival for the offspring. The extra attentional resources the parent spends on the anxiously-attached child are not wasted, however. One benefit the parents receive from their child's excessive attention-seeking behavior is that the child often remains home to assist the parents with the care of siblings. This type of “lineage reproduction” has been observed in birds (Emlen et al., 1995) and some human cultures (Clarke and Low, 1992).

### Health benefits

The ability to calibrate social pain may also have a profound array of physiological benefits. Chronic social rejection, in the form of loneliness and a lack of social support corresponds to a variety of physical health issues that are causally-linked to higher morbidity rates (see Cacioppo et al., 2003; Dickerson, 2011). A probable mechanism through which interpersonal isolation leads to such negative health outcomes is the ability of the social pain system to lead to suppress the immune system. Both the ACC and insula have strong functional connectivity with the hypothalamus, which forms the central nucleus of the hypothalamic-pituitary-adrenal axis (HPA axis; Davidson and Irwin, 1999; Eisenberger et al., 2007). The HPA axis partially functions to release cortisol throughout the bloodstream, which in many cases is an adaptive response to stressors, mobilizing the body to cope with threats. However, a side-effect of heightened cortisol is the suppression of the immune system. Eisenberger and colleagues (2007) explicated the neural mechanism through which social isolation causes immunosuppression, showing that not only is lowered dACC activation (in response to social threat) associated with lowered levels of endogenous cortisol, but that this association is mediated by the function of the hypothalamus. Put succinctly, dACC activation in response to social threat leads to recruitment of the hypothalamus which causes the release of cortisol which suppresses the immune system, which, finally, leads to negative health outcomes. While the authors did not test the effects of social rejection directly, these findings are likely to hold for this phenomenon as social rejection is one of the most prominent threats to our social selves.

Taylor and colleagues (2008) provided additional evidence for this process, initially reporting that greater psychosocial resources (i.e., support from friends and family) were associated with lower cortisol reactivity. Replicating previous work, ACC activation to a socially-threatening task was negatively associated with reported social support. More importantly, they found that psychosocial resources were positively correlated with recruitment of the right ventrolateral prefrontal cortex (rVLPFC) during a socially-threatening task, a neural region previously associated with the inhibitory regulation of social pain (Eisenberger et al., 2003). The association between psychosocial resources and reduced cortisol reactivity was mediated by activation of the rVLPFC. Interestingly, greater levels of psychosocial support did not predict decreases in amygdala activation in response to the social threat (Vrtièka et al., 2008), a brain region prominently associated with the detection of and response to threats. Taken together, these findings demonstrate that, among adults, social bonds facilitate the inhibition of the deleterious, physiological effects of social threat (i.e., immunosuppression via cortisol). However, adult social attachments did not affect the reactivity of areas associated with the detection of and psychological response to social threat. This last finding provides support that social pain calibration occurs in early life history stages, as predicted by the optimal calibration hypothesis.

For individuals chronically deprived of social bonds, such as avoidantly-attached persons, they would experience constant suppression of their immune system due to heightened endogenous cortisol levels. It would then make evolutionary sense that natural selection would down-calibrate social pain responses to chronic social rejection to avoid this potentially life-threatening health problem.

### Mating strategies

We know how the different attachment styles are beneficial in various types of childhood environments but as life history progresses, do they continue to be adaptive as mating strategies? Individuals high in avoidant attachment pursue a “quantity” rather than “quality” reproductive strategy in which they would invest in mating over parenting (Chisholm, 1996). Given the unpredictability of resources and care from their parents, survival was difficult, thus adopting a “fast” life history strategy in which avoidant individuals adopt a short-term mating strategy would be more adaptive given the likelihood of a shorter lifespan (see Del Giudice, 2009). Avoidant individuals' short-term mating strategy may indeed allow for the dispersal of genes that promote the down-calibration of the social pain network when childhood rejection appears chronic.

People high in anxious attachment are hypervigilant about the availability of their mates (Main et al., 1985; Hazan and Shaver, 1987; Collins and Read, 1990). This is represented neurally by the exacerbated activation seen in the right amygdala, a brain region associated with responding to threat, that anxiously-attached individuals show in response to negative social evaluation (Vrtièka et al., 2008). The hyperactivation of their threat system is especially evident during stressful situations with their mate. Indeed, anxiously-attached people become more angry, hostile, and anxious during conflicts with their mate than securely-attached individuals (Simpson and Rholes, 1994). During conflict resolution with their mates, people high in anxious attachment reported greater anger and hostility toward their mates compared to people low in anxious attachment. They also displayed greater stress and anxiety and rated their mates and relationships less positively compared to people low in anxious attachment (Simpson et al., 1996). The anger and hostility is thought to punish the mate for their unavailability and convey a need for comfort (Bowlby, 1973). Whereas these strategies are not always effective, they may function to prevent mate defection and curry long-term investment.

## Life history stages and social pain

Because the social pain network is sensitive to human social ecology, it would make evolutionary sense that this network would track our shifting social priorities across our various developmental stages. At each stage, the social pain network should become more responsive to rejection from social partners of utmost relevance to a given stage.

The evolution of life entails fundamental trade-offs in the allocation of energy and resources between survival-enhancing and reproductive activities. Life history Theory provides a framework for understanding how natural selection shaped the schedule and duration of key stages of development in an organism's life for optimal allocation of energy to maximize fitness (i.e., produce the largest number of surviving offspring; Kaplan and Gangestad, 2005). Organisms allocate energy through three different activities: growth, maintenance, and reproduction (Gadgil and Bossert, 1970). Growth and maintenance impact fitness through future reproduction, which creates a trade-off between the allocation of energy for current reproduction versus future reproduction (Bell and Koufopanou, 1986). An organism's life history is a result of selective pressures for solving these trade-offs, maximizing the total allocations of energy to reproduction across the life span (Charnov, 1993).

The life history stages of human development are each marked by increased attention and desired interaction with a unique set of attachment figures. Life History stages are as follows (in order): infancy, childhood, emerging adulthood, and adulthood (Kaplan et al., 2000). Each stage is characterized by shifts in fitness-relevant goals and subsequently, attachment figures. During infancy and childhood, humans require parental investment. As human children enter adolescence, an attachment emphasis arises toward peers. In emerging adulthood, individuals become more interested in finding mates. And throughout adulthood, humans invest heavily into their children who exist in a prolonged state of vulnerability.

Early life history stages are indicative of the nature of later life history stages. As such, experiences in infancy and childhood can alter adolescent and adult psychological processes. For individuals from uncertain and risky childhood environments, it would make evolutionary sense that, as adults, they would respond to such threats in an experienced and functional manner. In a brilliant series of experiments, Griskevicius et al. (2011) demonstrated that individuals from childhood environments characterized by scarcity and uncertainty (i.e., low socio-economic status) responded to reminders of their mortality by adaptively shifting their reproductive strategies toward the short-term. We argue that a similar process occurs in regards to social pain.

Interacting with a new set of potential attachment figures at each stage of development should have implications for how people experience social pain. That is, experiencing an episode of social rejection from an attachment figure who is particularly relevant to one's current life stage should evoke a stronger, more painful response than experiencing rejection from an attachment figure who is not particularly relevant to one's current life stage.

Thus, parental rejection should evoke a stronger response among infants and children than rejection from other attachment figures. Likewise, as people enter adolescence, peer rejection should elicit the strongest social pain response. In emerging adulthood, they should be especially sensitive to intimate partner rejection and concerned about guarding their mates. Last, adults who invest heavily into their offspring's welfare should be sensitive to episodes of rejection and rebellion from their children.

## Summary and future directions

Humans possess a powerful need to belong. A network of brain regions, centrally the dACC and anterior insula, evolved to elicit pain during experiences of social loss, threat, and rejection. The sensitivity of this system diverges according to anxious and avoidant attachment, with avoidant individuals showing reduced and anxious individuals showing increased activation of the social pain network in response to rejection. Adopting an evolutionary framework, we propose the optimal calibration hypothesis, which asserts that the frequency and intensity of rejection in early life history stages alters the sensitivity of the social pain system toward an adaptive level. Specifically, individuals who experience chronic rejection early in life should demonstrate a less sensitive social pain network, while individuals who experience volatile rejection early in life should possess a more sensitive social pain network.

Our hypothesis fits with previous research on attachment styles, which shows that avoidant individuals become desensitized toward social threat whereas anxious individuals become hypersensitive. The social pain network's plasticity likely evolved due to the fitness benefits that are commensurate with a flexible response to rejection. Individuals with a flexible social pain system are able to maintain social pain as an informative signal, recruit parental investment, retain and acquire mates, and avoid the health issues that are comorbid with social rejection.

Finally, we hypothesize that the social pain network should respond greater to rejecters of particular importance to a given life history stage. Given that human priorities shift across developmental trajectories, the need we have for specific types of social bonds (e.g., mates, caregivers) change in turn. As such, relationships that are more important at any given developmental stage should elicit greater activation in the social pain network when they are threatened.

Whereas the name of our theoretical approach is the optimal calibration hypothesis, we are fully aware that insecure attachment is not always an ideal social trajectory for individuals. We assert that the calibration of the social pain network is optimal in that it is the best possible outcome for individuals who must trade-off certain goals given a difficult early environment. These tradeoffs do not often lead to ideal life outcomes, but the benefits we outline in our paper certainly outweigh the disadvantages individuals would have to endure if their social pain systems were not adaptively calibrated to their early social ecology.

In this paper, we have hopefully provided interested scholars with several testable hypotheses. Future research may test our principal hypothesis by imaging the developing brains of individuals from early social environments characterized by chronic and volatile rejection. Our assertions that rejecters of utmost importance to a given life history stage will elicit greater social pain activation should also be put to the scientific test.

If empirical evidence is found for the optimal calibration hypothesis and its tenets, these findings would have implications for research on learned helplessness, a seemingly unrelated area of psychological inquiry. Learned helplessness is a phenomenon in which individuals placed in a painful environment wherein they can neither predict nor control the painful stimulus eventually cease their attempts at avoiding it (see Maier and Seligman, 1976). One could not find a better example of such an environment than that of the childhood of the insecurely-attached individual, in which they are repeatedly neglected by their caregivers for reasons outside of their control. While learned helplessness is rightfully portrayed as a maladaptive response, the tenets of the optimal calibration hypothesis would argue that the acceptance of such an environment is, in fact, a functional reaction. By accepting the likelihood of the continued nature of rejection from their caregivers, anxiously- and avoidantly-attached individuals can effectively calibrate their social pain networks to respond in an optimal fashion to future instances of rejection. This potential re-conceptualization of learned helplessness as occasionally adaptive would have substantial implications for the treatment and therapy of various mood and developmental disorders.

By conceptualizing the social pain network as a malleable substrate that responds adaptively to shifts in social ecology, we can better direct future research and treatment relating to social rejection. It is our hope that the framework of the optimal calibration hypothesis spurs on such advances.

### Conflict of interest statement

The authors declare that the research was conducted in the absence of any commercial or financial relationships that could be construed as a potential conflict of interest.
